# Association of subjective and objective physical activity with home hypertension

**DOI:** 10.1038/s41440-026-02587-8

**Published:** 2026-02-24

**Authors:** Saki Hayashi, Mana Kogure, Ippei Chiba, Rieko Hatanaka, Kumi Nakaya, Masato Takase, Sayuri Tokioka, Masatsugu Orui, Eiichi N. Kodama, Yohei Hamanaka, Mami Ishikuro, Taku Obara, Satoshi Nagaie, Tomohiro Nakamura, Soichi Ogishima, Sho Nagayoshi, Mitsuo Kuwabara, Toshiyuki Iwaoka, Nobuo Fuse, Yoko Izumi, Naoki Nakaya, Shinichi Kuriyama, Atsushi Hozawa

**Affiliations:** 1 OMRON Healthcare Co., Ltd., Kyoto, Japan; 2 Tohoku Medical Megabank Organization, Tohoku University, Sendai, Japan; 3 Graduate School of Medicine, Tohoku University, Sendai, Japan; 4 International Research Institute of Disaster Science, Tohoku University, Sendai, Japan; 5 Faculty of Data Science, Kyoto Women’s University, Kyoto, Japan

**Keywords:** Home blood pressure, Morning hypertension, Physical activity, Accelerometer, Physical activity questionnaire

## Abstract

Prevention of hypertension (HT), a risk factor for cardiovascular diseases, and blood pressure (BP) control are important. For the prevention and management of high BP, increased physical activity (PA) is recommended as a lifestyle intervention. Although various PA assessment methods exist, their associations with clinical BP have been inconsistent. This study aimed to compare self-reported and accelerometer-measured PA in relation to home HT prevalence based on home BP, which has better reproducibility than office BP. We conducted this cross-sectional study of 5895 participants (mean age: 57.5 years, 70.4% women) in the Tohoku Medical Megabank Project Cohort Study. Total PA was assessed using two methods: self-reported activities (leisure, occupational/household) and accelerometer-measured values. Home HT was defined as morning home BP ≥ 135/85 mmHg or under HT treatment. Modified Poisson regression analysis showed no statistically significant association between self-reported total PA and the prevalence of home HT. In contrast, higher levels of accelerometer-measured total PA were associated with lower prevalence of home HT (*P* for trend <0.05). Regarding other accelerometer-measured components, higher light PA and more steps were also significantly associated with lower prevalence of home HT. These associations were largely mediated by body mass index. In conclusion, accelerometer-measured PA, unlike self-reported PA, was associated with home HT, suggesting that PA assessed by accelerometers is useful for understanding the relationship between PA and HT, preventing HT, and managing high BP.

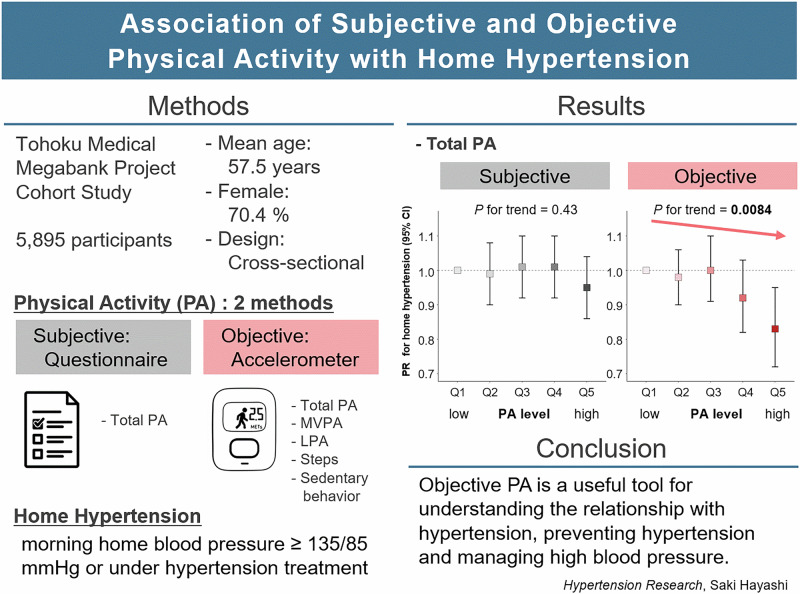

## Introduction

Hypertension (HT) is a risk factor for cardiovascular diseases, affecting an estimated 1.3 billion adults worldwide in 2019. An increase in the percentage of people with HT under control to 50% would prevent 76 million deaths between 2023 and 2050, but currently, only 21% of individuals have their blood pressure (BP) under control [1]. Therefore, preventing HT and controlling BP are crucial.

Lower physical activity (PA) is a risk factor for HT [2, 3]. HT practice guidelines recommend incorporating regular exercise and PA into daily life and reducing sedentary behavior (SB) as lifestyle interventions [2–5]. Thus, increasing PA is important for HT prevention and BP control.

Appropriate assessment of individual PA is important for understanding the relationship between PA and health as well as determining intervention effectiveness [6]. Free-living PA is evaluated via subjective (e.g., questionnaires) and objective (e.g., accelerometers) methods. Questionnaires offer higher feasibility than objective measurement, but their accuracy is limited [7]. Correlations between questionnaire and accelerometer data are weak to moderate [8]. A few large-scale studies have directly compared associations between PA assessed by questionnaires and accelerometers and health outcomes, such as HT or BP [9–13]. The results of these studies have been inconsistent, indicating uncertainty about whether different methods of PA assessment have distinct associations with HT and BP. Given this inconsistency, the preferred PA evaluation in clinical and public health contexts remains unclear. A potential reason for these inconsistent findings is their reliance on office BP. Home BP has better reproducibility than office BP [14] and is recommended in international guidelines for managing and diagnosing HT [2–5]; however, it remains unknown which PA assessment method (subjective or objective) is more strongly associated with home HT. Furthermore, objective measurement using accelerometers allows for a detailed assessment of PA components, such as moderate- to vigorous-intensity PA (MVPA), light-intensity PA (LPA), and SB, as well as steps. Understanding which components are strongly associated with home HT is crucial for developing targeted public health recommendations.

Therefore, this study primarily aimed to investigate the association between total PA evaluated using two methods—self-reported and accelerometer-measured—and the prevalence of home HT and secondarily aimed to assess the association of various accelerometer-measured components of total PA (MVPA, LPA, and SB) and steps with the prevalence of home HT.

Point of view

- Clinical relevance:
  Accelerometer-measured total physical activity, light-intensity physical activity, and steps are more strongly associated with a lower prevalence of home hypertension than self-reported measures. Therefore, physical activity monitoring with accelerometers could be useful for preventing hypertension and managing high blood pressure.
- Future direction:
  Intervention studies are required to confirm whether objective physical activity monitoring effectively prevents hypertension and improves blood pressure control.
- Consideration for the Asian population:
  Rapidly urbanizing Asian populations are facing lifestyle shifts similar to those observed in Japan. Given these environmental changes, assessing physical activity by accelerometers and increasing daily life activities may provide a culturally appropriate approach to reducing home hypertension prevalence across the region.

## Methods

### Participant recruitment

This cross-sectional study used data from the Tohoku Medical Megabank (TMM) Project Cohort Study [15–17]. Participants from the baseline survey (May 2013–March 2016) of the TMM Cohort Study were invited for a secondary assessment (June 2017–March 2021) and visited one of the community support centers. In June 2017, a collaborative study with OMRON Healthcare Co., Ltd. was started with participants from four centers within Miyagi prefecture.

By March 2021, 34,315 participants were enrolled in the secondary assessment. Of these, 18,842 were eligible for the collaborative study, and 9355 agreed to participate (as of November 10, 2024). All participants provided written informed consent. This study was approved by the Institutional Review Board of the TMM organization (approval numbers: 2017-4-007 and 2024-4-029).

### Inclusion/exclusion criteria

Of the 9355 initially enrolled participants, we first excluded 876 participants who visited centers without accelerometer distribution, leaving 8479 participants. From this group, we sequentially excluded participants for the following reasons: (1) valid accelerometer data for <4 days [18, 19] (*n* = 478; remaining *n* = 8001); (2) morning home BP measurements for <3 days [20] (*n* = 206; remaining *n* = 7795); (3) morning urinary sodium-to-potassium (Na/K) ratio measurements for <3 days [21] (*n* = 291; remaining *n* = 7504); (4) missing questionnaire data on PA, sex, age, weight, height, drinking status, smoking status, household income and history of diseases (*n* = 733; remaining *n* = 6771); and (5) history of cardiovascular diseases (i.e., cerebral hemorrhage, cerebral infarction, subarachnoid hemorrhage, myocardial infarction, angina pectoris, aneurysm, aortic dissection, heart failure, Marfan syndrome, atrial fibrillation, ventricular fibrillation, presence of a pacemaker, or presence of an implantable cardioverter-defibrillator), dementia, rheumatism, and chronic obstructive pulmonary disease (*n* = 876). The final sample included 5895 participants.

### Measurement

#### Self-reported PA

The participants reported their activities (sleep, leisure, occupational/household) over the last year via a questionnaire (validated for total PA [22]). Specifically, this questionnaire asked the participants to report the daily duration of occupational/household activities at four different intensity levels (sitting, standing, walking, and strenuous work). For leisure activities, it assessed the frequency and duration at four intensity levels (walking slowly, walking quickly, light-to-moderate exercise, and strenuous exercise). Regarding sleep, the participants were asked a single question: “On average, how many hours do you sleep per day?” Total PA was calculated by multiplying the sum of time spent on each activity, excluding sleep, by the metabolic equivalents (METs) of the assigned task. MVPA was the sum of activities with intensity ≥3.0 METs. Reporting times exceeding 24 h were corrected by multiplying by 24/total reported time. Self-reported PA is denoted as PA-SR, and self-reported total PA is referred to as total PA-SR.

#### Accelerometer-measured PA

A triaxial accelerometer (Active style Pro HJA-750C; OMRON Healthcare Co., Ltd., Kyoto, Japan, a previously validated device [23, 24]), worn on the waist (excluding sleep and underwater activities) for 10 consecutive days, assessed MVPA, LPA, SB, steps, in addition to total PA. The first day’s data were excluded. To process the accelerometer data, we used a macro program from the Japan Physical Activity Research Platform [25]. 60-s epoch length data were used. Non-wear was defined by periods of at least 60 consecutive minutes with no detected activity signal, allowing for interruptions of 1 or 2 min with <1.0 METs. Valid data required ≥600 min of total wear time [19]. PA intensity was categorized by METs: ≤1.5 for SB, 1.6–2.9 for LPA, and ≥3.0 for MVPA. Total PA was the sum of METs-hours from all daily activities (METs-h/d). Accelerometer-measured PA is denoted as PA-Acc, and accelerometer-measured total PA is referred to as total PA-Acc.

#### Home BP and HT definition

Home BP was measured using a BP monitor (HEM-7080IC; OMRON Healthcare Co., Ltd., Kyoto, Japan) for 10 days during the same period as PA measurement. Participants measured morning BP within 1 h of waking, after urination, and before taking medication or eating breakfast. Morning home BP was defined as the mean of the first and second measurements each morning, calculated across ≥3 days.

Home HT was defined as morning home systolic BP ≥ 135 mmHg and/or morning home diastolic BP ≥ 85 mmHg or currently receiving treatment. HT treatment status was self-reported via a questionnaire as one of the following options: (1) previously diagnosed with HT (currently receiving treatment, discontinued treatment, managed with lifestyle changes without medication, or under observation without medication) or (2) never diagnosed with HT.

#### Measurements for the covariates

Covariate selection was based on previous epidemiological evidence and clinical relevance of factors associated with PA and BP [1–5, 26]. Potential confounders included sociodemographic variables (age, sex, household income), lifestyle factors (body mass index (BMI), drinking, smoking, urinary Na/K ratio), accelerometer wear time, and seasonality. Household income was categorized into four groups: less than 2 million yen, 2 to <4 million yen, 4 to <6 million yen, and 6 million yen or more. BMI was calculated by dividing weight (kg) by height (m) squared. Drinking status was categorized into three groups: never-drinker, past-drinker, and current-drinker. Smoking status was categorized into three groups: never-smoker (smoked fewer than 100 cigarettes in their lifetime), past-smoker (smoked 100 or more cigarettes in their lifetime but was not currently smoking), and current-smoker. Urinary Na/K ratio was measured twice daily (morning and evening) using a hand-sized urinary Na/K ratio monitor (HEU-001F; OMRON Healthcare Co., Ltd., Kyoto, Japan) for 10 days during the same period as PA measurement. The average morning measurements were used because the morning urinary Na/K ratio is linearly associated with morning home BP [21]. When multiple measurements were taken in the same morning (4:00–11:00 AM), only the first was used. We hypothesized that the morning urinary Na/K ratio is associated with BP (outcome) and PA (exposure) through common causes such as overall dietary patterns or health awareness, rather than acting as a mediator. This is supported by previous findings showing lower PA levels in the group with a high Na/K ratio [27] and reporting no significant association between PA levels and the urinary sodium excretion circadian rhythm [28]. Seasons were classified by average monthly temperatures in Sendai City (2016–2018), representing Miyagi Prefecture. The period of June–September was defined as summer, December–March as winter, and the remaining months as other seasons [29].

### Statistical analysis

Continuous variables are presented as mean (standard deviation [SD]) and categorical variables as *n* (%). Trend tests evaluated linear relationships between PA and participants’ characteristics. General linear and logistic regression models were used for continuous and categorical variables, respectively. We evaluated the association between total PA-SR and total PA-Acc using multivariate linear regression models, adjusted for sex, age, and household income, and Spearman’s rank correlation analysis.

The participants were classified into five groups by quintiles of total PA-SR, total PA-Acc, MVPA, LPA, SB, and steps, respectively. Modified Poisson regression models were used to obtain the prevalence ratio (PR) and 95% confidence interval (CI) for the prevalence of home HT by PA quintile. Adjusted PR and *P*-values for linear trends (*P* for trend) were calculated using PA quintiles; <0.05 was considered statistically significant.

Model 1 included the PA metric and total wear time (only PA-Acc) to examine crude associations. Model 2 was further adjusted for sociodemographic characteristics and seasonality. Model 3 was further adjusted for drinking, smoking, and urinary Na/K ratio. Model 4 was further adjusted for BMI. Given that BMI could be an intermediate variable or a mediator, Models 1, 2, and 3 treated BMI as a mediator and did not adjust for it. Model 4 was used to assess whether BMI acts as an intermediate variable in the relationship between PA and HT.

Additionally, as a sensitivity analysis, to eliminate the influence of HT treatment, we excluded participants receiving treatment for HT (*n* = 1200) and examined the association between total PA and home HT. All analyses were performed using software R (ver.4.1.2).

## Results

Table 1 shows the baseline characteristics of the participants categorized by total PA-Acc. The mean age of the participants was 57.5 years, with more than half (70.4%, *n* = 4151) being women and 2296 (38.9%) having home HT. Those with longer total wear times were more likely to have increased total PA-Acc (*P* for trend < 0.001). Conversely, older age, high BMI, current drinking, and current smoking were associated with decreased total PA-Acc (*P* for trend < 0.001). Participants who measured in summer tended to show higher total PA-Acc, and those who measured in winter showed lower total PA-Acc (*P* for trend < 0.001). Morning urinary Na/K ratio was negatively associated with total PA-Acc (*P* for trend = 0.033). Additional details on baseline characteristics categorized by other PA metrics are provided in the online supplemental Tables (Supplementary Tables 1–5). Spearman’s correlation coefficient between total PA-SR and total PA-Acc was 0.24 (*P* < 0.001) (data not shown). Multiple regression analysis with total PA-SR as the objective variable and total PA-Acc as the explanatory variable, adjusted for sex, age, and household income, showed significant associations for total PA-Acc (*P* < 0.001) (Supplementary Table 6).

**Table 1:** Baseline characteristics of the participants according to total PA-Acc

		Overall	Total PA-Acc					*P* for trend
			Q1	Q2	Q3	Q4	Q5	
Participants, *n*		5895	1179	1179	1180	1178	1179	
Age (years)		57.5 (14.1)	61.0 (14.7)	58.2 (14.8)	56.4 (14.7)	56.1 (13.3)	55.8 (12.0)	<0.001
Sex	Men	29.6 (1744)	58.4 (688)	36.2 (427)	24.2 (285)	15.8 (186)	13.4 (158)	<0.001
BMI (kg/m^2^)		23.1 (3.4)	24.1 (3.5)	23.6 (3.6)	23.0 (3.3)	22.7 (3.2)	22.3 (3.1)	<0.001
Morning home SBP (mmHg)		125.0 (16.9)	129.1 (17.2)	126.7 (17.1)	124.1 (16.6)	122.6 (16.1)	122.3 (16.3)	<0.001
Morning home DBP (mmHg)		75.0 (10.1)	76.8 (10.3)	75.7 (10.2)	74.6 (9.7)	74.2 (9.8)	73.6 (10.2)	<0.001
Home HT^a^	Yes	38.9 (2296)	52.4 (618)	42.8 (505)	38.1 (450)	32.9 (388)	28.4 (335)	<0.001
Treatment for HT	Yes	20.4 (1200)	30.8 (363)	23.3 (275)	19.1 (225)	15.6 (184)	13.0 (153)	<0.001
Household income	<2 million yen	11.6 (684)	14.8 (175)	11.7 (138)	13.0 (153)	10.1 (119)	8.4 (99)	<0.001
	2 to <4 million yen	39.3 (2316)	42.2 (497)	41.4 (488)	38.1 (449)	37.3 (439)	37.6 (443)	0.0032
	4 to <6 million yen	23.7 (1400)	21.8 (257)	22.4 (264)	24.1 (284)	25.6 (301)	24.9 (294)	0.016
	≥6 million yen	25.4 (1495)	21.2 (250)	24.5 (289)	24.9 (294)	27.1 (319)	29.1 (343)	<0.001
Seasonality	Summer	38.7 (2279)	35.3 (416)	34.9 (412)	37.6 (444)	41.9 (494)	43.5 (513)	<0.001
	Winter	33.0 (1944)	39.2 (462)	34.6 (408)	33.3 (393)	31.1 (366)	26.7 (315)	<0.001
	Other	28.4 (1672)	25.5 (301)	30.4 (359)	29.1 (343)	27.0 (318)	29.8 (351)	0.23
Drinking status	Never	48.5 (2861)	40.5 (477)	43.9 (518)	52.6 (621)	53.6 (631)	52.1 (614)	<0.001
	Past	2.3 (138)	3.3 (39)	2.7 (32)	2.3 (27)	1.8 (21)	1.6 (19)	0.0021
	Current	49.1 (2896)	56.2 (663)	53.4 (629)	45.1 (532)	44.7 (526)	46.3 (546)	<0.001
Smoking status	Never	66.0 (3889)	48.3 (570)	62.1 (732)	69.9 (825)	74.0 (872)	75.5 (890)	<0.001
	Past	25.9 (1526)	39.1 (461)	28.5 (336)	23.6 (278)	20.0 (236)	18.2 (215)	<0.001
	Current	8.1 (480)	12.6 (148)	9.4 (111)	6.5 (77)	5.9 (70)	6.3 (74)	<0.001
Morning urinary Na/K ratio		4.7 (1.9)	4.9 (2.0)	4.8 (2.0)	4.6 (1.8)	4.7 (2.0)	4.7 (2.0)	0.033
Total wear time (min/day)		907.9 (95.7)	808.8 (69.7)	870.2 (65.3)	907.5 (66.9)	951.4 (67.5)	1001.5 (75.9)	<0.001
Total PA-Acc (METs-h/day)		25.9 (3.9)	20.7 (1.6)	23.8 (0.6)	25.8 (0.6)	27.9 (0.7)	31.4 (2.1)	<0.001
Total PA-SR (METs-h/day)		41.4 (13.7)	36.9 (11.9)	39.9 (12.8)	41.5 (13.5)	43.0 (13.8)	46.0 (14.7)	<0.001
MVPA (min/day)		61.1 (34.9)	34.2 (18.6)	47.9 (20.8)	56.8 (24.6)	67.8 (25.9)	98.7 (40.7)	<0.001
LPA (min/day)		385.3 (95.6)	274.6 (63.4)	343.3 (57.5)	389.8 (56.0)	429.7 (59.1)	489.1 (70.7)	<0.001
SB (min/day)		461.5 (110.0)	500.0 (105.5)	479.0 (108.0)	460.9 (106.0)	454.0 (106.5)	413.7 (105.0)	<0.001
Steps (steps/day)		6178.9 (2728.7)	4387.9 (2011.6)	5535.8 (2214.9)	6021.5 (2265.8)	6724.2 (2408.6)	8225.6 (3037.7)	<0.001

*BMI* body mass index, *SBP* systolic blood pressure, *DBP* diastolic blood pressure, *HT* hypertension, *total PA* total physical activity, *METs* metabolic equivalents, *MVPA* moderate- to vigorous-intensity physical activity, *SB* sedentary behavior, *LPA* light-intensity physical activity, *Acc* accelerometer-measured, *SR* self-reported, *Na/K ratio* Sodium-to-potassium ratio

^a^Home HT was defined as morning home SBP ≥ 135 mmHg and/or DBP ≥ 85 mmHg or receiving treatment for hypertension

### Association between PA and prevalence of home HT

The association between total PA and the prevalence of home HT is shown in Table 2. No significant linear association was observed between total PA-SR and home HT in any model. Total PA-Acc showed a significant inverse association with the prevalence of home HT in Model 1 (*P* for trend < 0.001), Model 2 (*P* for trend = 0.020), and Model 3 (*P* for trend = 0.0084). After adjustment for BMI, this association was attenuated, suggesting that BMI acts as an intermediate variable (*P* for trend = 0.82).

**Table 2:** Association between total PA-SR or total PA-Acc and the prevalence of home HT among all participants

	Model 1^a^		Model 2^b^		Model 3^c^		Model 4^d^	
	PR (95% CI)	*P* for trend	PR (95% CI)	*P* for trend	PR (95% CI)	*P* for trend	PR (95% CI)	*P* for trend
Total PA-SR (METs-h/day)								
Q1 (26.5 (1.8))	1.00 (ref.)	0.39	1.00 (ref.)	0.73	1.00 (ref.)	0.43	1.00 (ref.)	0.85
Q2 (31.6 (1.7))	0.97 (0.87–1.07)		0.98 (0.89–1.08)		0.99 (0.90–1.08)		1.02 (0.93–1.11)	
Q3 (37.7 (2.0))	1.09 (0.98–1.20)		1.01 (0.92–1.10)		1.01 (0.92–1.10)		1.02 (0.94–1.12)	
Q4 (47.6 (3.6))	1.08 (0.98–1.19)		1.01 (0.92–1.10)		1.01 (0.92–1.10)		1.06 (0.97–1.15)	
Q5 (63.8 (6.5))	**0.90 (0.81–0.996)**		0.97 (0.88–1.06)		0.95 (0.86–1.04)		0.99 (0.90–1.08)	
Total PA-Acc (METs-h/day)								
Q1 (20.7 (1.6))	1.00 (ref.)	**<0.001**	1.00 (ref.)	**0.020**	1.00 (ref.)	**0.0084**	1.00 (ref.)	0.82
Q2 (23.8 (0.6))	**0.82 (0.75–0.90)**		0.98 (0.90–1.07)		0.98 (0.90–1.06)		1.01 (0.93–1.10)	
Q3 (25.8 (0.6))	**0.73 (0.66–0.81)**		0.99 (0.90–1.09)		1.00 (0.91–1.10)		1.08 (0.99–1.19)	
Q4 (27.9 (0.7))	**0.64 (0.57–0.72)**		0.93 (0.83–1.04)		0.92 (0.82–1.03)		1.05 (0.94–1.17)	
Q5 (31.4 (2.1))	**0.55 (0.48–0.63)**		**0.85 (0.74–0.97)**		**0.83 (0.72–0.95)**		0.99 (0.87–1.13)	

Models 1–3: Unadjusted for BMI (as a mediator). Model 4: Adjusted for BMI (to assess whether BMI acts as an intermediate variable)

*HT* hypertension, *BMI* body mass index, *total PA* total physical activity, *METs* metabolic equivalents, *PR* prevalence ratio, *CI* confidence interval, *Acc* accelerometer-measured, *SR* self-reported, *Na/K ratio* Sodium-to-potassium ratio

^a^Adjusted for total wear time (only accelerometer-measured)

^b^Adjusted for variables in Model 1 + age, sex, household income, and seasonality

^c^Adjusted for variables in Model 2 + drinking status, smoking status, and urinary Na/K ratio

^d^Adjusted for variables in Model 3 + BMI

Bold values indicate statistical significance (*P* < 0.05 or *P* for trend < 0.05).

In the sensitivity analysis, we excluded participants receiving treatment for HT (Table 3). Similar to the main results, total PA-SR was not linearly associated with the prevalence of home HT. For total PA-Acc, although the association was not significant in Model 2 (*P* for trend = 0.62) and Model 3 (*P* for trend = 0.27) (while remaining significant in Model 1; *P* for trend < 0.001), the point estimate of the PR for the highest quintile (Q5) compared to the lowest (Q1) in Model 3 was 0.86, similar to the results of the main analysis (0.83).

**Table 3:** Association between total PA-SR or total PA-Acc and the prevalence of home HT among participants not receiving treatment for HT

	Model 1^a^		Model 2^b^		Model 3^c^		Model 4^d^	
	PR (95% CI)	*P* for trend	PR (95% CI)	*P* for trend	PR (95% CI)	*P* for trend	PR (95% CI)	*P* for trend
Total PA-SR (METs-h/day)								
Q1 (26.6 (1.8))	1.00 (ref.)	0.44	1.00 (ref.)	0.50	1.00 (ref.)	0.83	1.00 (ref.)	0.85
Q2 (31.6 (1.7))	1.11 (0.94–1.32)		1.10 (0.93–1.29)		1.09 (0.93–1.27)		1.11 (0.95–1.30)	
Q3 (37.7 (2.0))	1.14 (0.96–1.35)		1.06 (0.90–1.25)		1.04 (0.88–1.22)		1.04 (0.89–1.22)	
Q4 (47.6 (3.6))	1.25 (1.06–1.47)		1.14 (0.98–1.34)		1.09 (0.93–1.27)		1.12 (0.96–1.31)	
Q5 (63.9 (6.4))	1.02 (0.86–1.21)		1.04 (0.88–1.23)		0.98 (0.83–1.16)		1.01 (0.86–1.19)	
Total PA-Acc (METs-h/day)								
Q1 (20.8 (1.5))	1.00 (ref.)	**<0.001**	1.00 (ref.)	0.62	1.00 (ref.)	0.27	1.00 (ref.)	0.53
Q2 (23.8 (0.6))	**0.83 (0.71–0.97)**		1.00 (0.85–1.16)		0.97 (0.84–1.13)		1.02 (0.88–1.18)	
Q3 (25.8 (0.6))	**0.78 (0.65–0.92)**		1.07 (0.91–1.27)		1.06 (0.90–1.25)		1.17 (0.998–1.38)	
Q4 (27.9 (0.7))	**0.69 (0.56–0.83)**		1.02 (0.84–1.24)		0.98 (0.81–1.19)		1.14 (0.94–1.38)	
Q5 (31.4 (2.1))	**0.60 (0.48–0.76)**		0.92 (0.74–1.15)		0.86 (0.69–1.07)		1.02 (0.82–1.27)	

Models 1–3: Unadjusted for BMI (as a mediator). Model 4: Adjusted for BMI (to assess whether BMI acts as an intermediate variable)

*HT* hypertension, *BMI* body mass index, *total PA* total physical activity, *METs* metabolic equivalents, *PR* prevalence ratio, *CI* confidence interval, *Acc* accelerometer-measured, *SR* self-reported, *Na/K ratio* Sodium-to-potassium ratio

^a^Adjusted for total wear time (only accelerometer-measured)

^b^Adjusted for variables in Model 1 + age, sex, household income, and seasonality

^c^Adjusted for variables in Model 2 + drinking status, smoking status, and urinary Na/K ratio

^d^Adjusted for variables in Model 3 + BMI

Bold values indicate statistical significance (*P* < 0.05 or *P* for trend < 0.05).

Table 4 presents the association between accelerometer-measured components of total PA and the prevalence of home HT. MVPA and SB were not significantly associated with the prevalence of home HT except in the crude model. LPA was significantly inversely associated with the prevalence of home HT in Model 1 (*P* for trend < 0.001) and Model 3 (*P* for trend = 0.025). After adjustment for BMI, this association was attenuated, suggesting that BMI acts as an intermediate variable (*P* for trend = 0.77). Steps were significantly inversely associated with the prevalence of home HT in Model 1 (*P* for trend < 0.001), Model 2 (*P* for trend = 0.014), and Model 3 (*P* for trend = 0.042). Adjusting for BMI attenuated the relation, suggesting that BMI acts as an intermediate variable (*P* for trend = 0.62).

**Table 4:** Association of accelerometer-measured components of total PA (MVPA, LPA, SB) and steps with the prevalence of home HT among all participants

	Model 1^a^		Model 2^b^		Model 3^c^		Model 4^d^	
	PR (95% CI)	*P* for trend	PR (95% CI)	*P* for trend	PR (95% CI)	*P* for trend	PR (95% CI)	*P* for trend
MVPA (min/day)								
Q1 (22.6 (7.8))	1.00 (ref.)	**<0.001**	1.00 (ref.)	0.12	1.00 (ref.)	0.26	1.00 (ref.)	0.53
Q2 (40.6 (4.3))	**0.86 (0.78–0.94)**		1.00 (0.92–1.09)		1.02 (0.94–1.10)		1.04 (0.96–1.13)	
Q3 (55.0 (4.3))	**0.76 (0.69–0.84)**		0.98 (0.89–1.07)		1.00 (0.92–1.10)		1.05 (0.96–1.14)	
Q4 (72.6 (6.1))	**0.75 (0.68–0.82)**		0.92 (0.84–1.01)		0.93 (0.85–1.02)		0.99 (0.90–1.08)	
Q5 (114.8 (31.6))	**0.79 (0.72–0.87)**		0.96 (0.88–1.05)		0.98 (0.90–1.08)		1.06 (0.97–1.16)	
LPA (min/day)								
Q1 (249.6 (42.8))	1.00 (ref.)	**<0.001**	1.00(ref.)	0.13	1.00(ref.)	**0.025**	1.00(ref.)	0.77
Q2 (334.0 (16.6))	**0.82 (0.75–0.89)**		0.96(0.88–1.04)		0.95(0.88–1.03)		0.98(0.90–1.06)	
Q3 (387.2 (14.4))	**0.70 (0.64–0.77)**		0.96(0.88–1.06)		0.94(0.86–1.03)		1.01(0.92–1.10)	
Q4 (438.0 (16.2))	**0.67 (0.61–0.74)**		0.97(0.88–1.07)		0.95(0.86–1.04)		1.03(0.94–1.13)	
Q5 (517.6 (42.6))	**0.59 (0.52–0.66)**		0.89(0.79–1.0001)		**0.86 (0.77–0.97)**		0.99(0.88–1.10)	
SB (min/day)								
Q1 (313.0 (46.3))	1.00(ref.)	**<0.001**	1.00 (ref.)	0.16	1.00 (ref.)	0.054	1.00 (ref.)	0.37
Q2 (400.7 (18.7))	**1.14 (1.02–1.27)**		0.94 (0.85–1.04)		0.94 (0.85–1.04)		0.91 (0.83–1.0007)	
Q3 (457.6 (15.6))	**1.27 (1.14–1.41)**		0.98 (0.89–1.08)		0.99 (0.90–1.10)		0.94 (0.86–1.04)	
Q4 (517.2 (19.1))	**1.44 (1.29–1.60)**		1.00 (0.91–1.11)		1.02 (0.92–1.12)		0.93 (0.84–1.02)	
Q5 (619.3 (60.2))	**1.66 (1.49–1.85)**		1.05 (0.95–1.17)		1.07 (0.97–1.19)		0.93 (0.84–1.04)	
Steps (steps/day)								
Q1 (2971.9 (783.8))	1.00 (ref.)	**<0.001**	1.00 (ref.)	**0.014**	1.00 (ref.)	**0.042**	1.00 (ref.)	0.62
Q2 (4611.3 (361.0))	**0.81 (0.73–0.88)**		0.97 (0.89–1.06)		0.98 (0.90–1.07)		1.04 (0.96–1.13)	
Q3 (5799.0 (351.8))	**0.77 (0.70–0.85)**		0.93 (0.85–1.01)		0.93 (0.85–1.01)		1.02 (0.94–1.11)	
Q4 (7203.2 (480.6))	**0.75 (0.68–0.83)**		0.92 (0.84–1.01)		0.93 (0.84–1.01)		1.02 (0.93–1.12)	
Q5 (10309.0 (2265.9))	**0.77 (0.70–0.85)**		**0.91 (0.83–0.99)**		0.93 (0.85–1.02)		1.03 (0.94–1.13)	

Models 1–3: Unadjusted for BMI (as a mediator). Model 4: Adjusted for BMI (to assess whether BMI acts as an intermediate variable)

*HT* hypertension, *BMI* body mass index, *MVPA* moderate- to vigorous-intensity physical activity, *LPA* light-intensity physical activity, *SB* sedentary behavior, *PR* prevalence ratio, *CI* confidence interval, *Na/K ratio* Sodium-to-potassium ratio

^a^Adjusted for total wear time (only accelerometer-measured)

^b^Adjusted for variables in Model 1 + age, sex, household income, and seasonality

^c^Adjusted for variables in Model 2 + drinking status, smoking status, and urinary Na/K ratio

^d^Adjusted for variables in Model 3 + BMI

Bold values indicate statistical significance (*P* < 0.05 or *P* for trend < 0.05).

## Discussion

We investigated the association between PA evaluated using two methods—self-reported and accelerometer-measured—and the prevalence of home HT. This study revealed no significant association between total PA-SR and the prevalence of home HT. In contrast, increased total PA-Acc was associated with a lower prevalence of home HT. Regarding other accelerometer-measured components, higher LPA and more steps were also significantly associated with lower prevalence of home HT. The association between PA-Acc and home HT was substantially attenuated or disappeared after adjusting for BMI, suggesting that BMI acts as an intermediate variable.

Previous studies have evaluated the association of PA with BP value, prevalence of HT, or incidence of HT. Our finding that only PA-Acc, and not PA-SR, was associated with the prevalence of home HT aligns with the findings of several previous studies. Further focusing on the intensity of PA-Acc, the inverse association between LPA and home HT was consistent with the results of a study in another region of Japan, which reported that LPA was lower among men with HT [30]. However, we did not find a significant association between HT and MVPA. This finding is inconsistent with the results of three previous studies that reported a significant inverse association between MVPA and HT [9, 10, 13]. This discrepancy may be attributable to differences in study design and methodology. First, those previous studies used office BP [9] or defined HT based on office BP or medication use [13]. Office BP has lower reproducibility, which may introduce measurement variability. Second, confounding owing to comorbidities may explain the differing results. The handling of major comorbidities in those studies was inconsistent. For example, one study attempted to control for coronary artery disease via statistical adjustment rather than exclusion, leaving potential residual confounding [9]. Another study did not account for such comorbidities through either exclusion or adjustment [13]. This lack of exclusion, or in some cases insufficient adjustment, likely means that individuals with low PA owing to other chronic diseases remained in the HT group. This discrepancy may have magnified the difference in PA between the groups, potentially overestimating the association. Additionally, the context of PA (e.g., leisure vs. occupational), which our study did not assess, may be crucial. One study using ambulatory BP monitoring found that while objectively measured occupational MVPA showed no association, objectively measured high leisure MVPA was significantly associated with lower mean systolic BP during the daytime [10].

Our study found a stronger association between increased total PA-Acc and reduced prevalence of home HT than total PA-SR. A weak-to-moderate correlation between PA-SR and PA-Acc has been reported previously [8]. This discrepancy arises not only from distinct aspects of PA measured by different methods but also from errors in PA measurement caused by biases (e.g., social desirability, inaccurate memory) inherent in self-reporting [31]. Accelerometers have less variability in terms of validity and reliability than subjective methods [32]. Undoubtedly, PA-SR is an important option in epidemiological investigations owing to its high feasibility and cost-effectiveness. Although budgetary constraints may limit the use of accelerometers, our results suggest that using accelerometers may be the appropriate choice for understanding the association between PA level and health outcomes.

The association between PA-Acc and home HT was dependent on BMI. This association aligns with the practical value of monitoring body weight, which represents a simple and feasible target for HT management and prevention. However, this point does not reduce the importance of assessing PA, as PA is a modifiable upstream factor that influences BMI on the pathway to HT. Specifically, our study found that high LPA and an increased number of steps were associated with a low prevalence of home HT, an association mediated by BMI. Activities such as LPA and reducing SB are key components of non-exercise activity thermogenesis (NEAT), which consists of activities that do not involve MVPA and accounts for more than 15% of total daily energy expenditure. An increase in NEAT has been reported to reduce the occurrence of metabolic syndromes and cardiovascular events [33]. Therefore, our findings suggest that higher LPA and steps were associated with lower prevalence of home HT indirectly, by lowering BMI via an increase in NEAT. This highlights that all PA intensities, not just MVPA, are important components in the prevention and management of home HT.

This study has four strengths. First, morning home BP ≥ 135/85 mmHg was used to define home HT. Home BP better predicts cardiovascular risk than office BP [34]. Second, two different PA assessment methods were conducted simultaneously in the same population. Direct comparisons minimized the effect of differences in the timing of measurements and the population. These factors strengthened the reliability of the results. Third, we examined several components of PA metrics using the accelerometer, which excels in evaluating free-living PA [23], allowing for a detailed analysis of which PA intensities are most associated with home HT. The results suggest that higher total PA-Acc and its component, LPA, were associated with a lower prevalence of HT, highlighting their potential importance for BP control. This finding is consistent with that of a previous study involving older Japanese adults, which demonstrated that in populations with low MVPA (e.g., women), the total PA can be higher, driven primarily by the accumulation of LPA and short-bout MVPA, compared to that in men [35]. Additionally, given the seasonality and large individual differences [36, 37] in PA patterns, PA-Acc may be useful in personalized healthcare. Fourth, the robustness of our primary findings was supported by a sensitivity analysis that excluded participants receiving treatment for HT (*n* = 1200). In this subgroup, although the association for total PA-Acc lost statistical significance after full adjustment, except for BMI (Model 3, *P* for trend = 0.27; Table 3), the direction of the association and the point estimate for the highest quintile (PR = 0.86; Table 3) remained consistent with those obtained in our main analysis (PR = 0.83; Table 2). This loss of statistical significance is likely attributable to a decrease in statistical power, as the exclusion comprised 20.4% of the total participants (1200/5895) and represented 52.3% of all home HT cases (1200/2296).

However, this study has five limitations. First, the cross-sectional design precludes causal inference between PA and home HT. Intervention studies are needed to determine whether the PA level assessed by accelerometers can help prevent HT or improve BP control. Second, the participation rate in the collaborative study was 49.6%, and younger individuals were more likely to participate. Previous research showed that correlation coefficients between PA-SR and PA-Acc were lower among older individuals [38]. Consequently, this selection bias may underestimate the discrepancy between PA-SR and PA-Acc by excluding those with less accurate self-reporting. Third, caution is required when generalizing the results owing to the presence of selection bias. Specifically, participants from certain regions and those unable to provide valid device measurements were excluded. If the excluded individuals had both low PA and a high prevalence of HT, selection bias potentially led to an underestimation of the true association. Although this potential for bias must be acknowledged, it is noteworthy that the proportion of participants excluded due to insufficient valid accelerometer data (*n* = 478, 5.6%) was lower than the exclusion rates (over 20%) reported in similar large observational studies [9, 13]. Fourth, information on the stage of behavioral change regarding lifestyle modification was not available. Although it is possible that participants with higher PA levels were already in the action or maintenance stages, our sensitivity analysis that excluded participants receiving treatment for HT—who are presumed to be receiving medical advice for lifestyle modification—showed the robustness of our main findings, suggesting that this limitation was unlikely to have substantially biased the results. Fifth, although this study demonstrates the advantages of accelerometer measurements, practical barriers exist to their application as effective intervention tools. For example, long-term user adherence remains a challenge and requires further investigation.

The Japanese Society of Hypertension 2025 guidelines emphasize aerobic and resistance exercise [5]. In addition to these structured exercises, PA includes “daily life activities” (e.g., household, work, and commuting) that contribute to NEAT. Our study highlights the importance of objectively capturing PA, as we found that total PA-Acc and LPA, a key component of NEAT, were significantly associated with a lower prevalence of home HT. These associations were largely mediated by BMI.

While these findings demonstrate the benefits of PA, the context of PA varies significantly due to differences in socioeconomic circumstances and levels of urbanization [39]. As rapid urbanization progresses throughout Asia, lifestyle environments are increasingly resembling those of Japan. Consequently, our findings in Japan suggest that increasing daily life activities provides a culturally applicable strategy in Asia. To our knowledge, this is the first report in an Asian population to compare subjective and objective PA assessments in relation to home HT, and further replication studies in other Asian regions are needed.

In conclusion, a clear discrepancy was found between measurement methods: accelerometer-measured total PA was associated with home HT, unlike self-reported total PA, highlighting the importance of the measurement method when assessing the association with health outcomes like HT. Among other accelerometer-measured metrics, higher LPA and steps were also significantly associated with lower prevalence of home HT. These associations were largely mediated by BMI. For effective prevention, it is crucial not only to focus on strong factors like BMI but also to accurately capture upstream lifestyle factors like PA. Given the large individual differences in PA patterns, objective data from accelerometers, which capture LPA and SB that are often self-reported inaccurately, could be a useful tool to prevent HT and manage high BP.

## Supplementary information

Supplementary information

Supplementary Table 1

Supplementary Table 2

Supplementary Table 3

Supplementary Table 4

Supplementary Table 5

Supplementary Table 6
