# Supplementary material for: Association of subjective and objective physical activity with home hypertension
Source: Hypertens Res. 2026 Feb 24;49(5):1586–96. doi: 10.1038/s41440-026-02587-8 (PMC13148978; doi:10.1038/s41440-026-02587-8)
Supplement: Supplementary file 1 — Supplementary information [file 41440_2026_2587_MOESM1_ESM.docx]

All supplementary files(.docx) are cited in the text.

---

Supplementary Table 1：The baseline characteristics of the participants categorized by self-reported total physical activity

Supplementary Table 2：The baseline characteristics of the participants categorized by moderate-vigorous physical activity

Supplementary Table 3：The baseline characteristics of the participants categorized by light physical activity

Supplementary Table 4：The baseline characteristics of the participants categorized by sedentary behavior

Supplementary Table 5：The baseline characteristics of the participants categorized by steps

Supplementary Table 6：Relationship evaluation by multiple regression analysis with self-reported total physical activity as the objective variable and accelerometer-measured total physical activity as the explanatory variable, adjusted for sex, age and household income
