# Supplementary material for: Association of subjective and objective physical activity with home hypertension
Source: Hypertens Res. 2026 Feb 24;49(5):1586–96. doi: 10.1038/s41440-026-02587-8 (PMC13148978; doi:10.1038/s41440-026-02587-8)
Supplement: Supplementary file 2 — Supplementary Table 1 [file 41440_2026_2587_MOESM2_ESM.docx]

**Supplementary Table 1: Baseline characteristics of the participants according to total PA-SR**

|  |  | Overall | Total PA-SR |  |  |  |  | *P* for trend |
| --- | --- | --- | --- | --- | --- | --- | --- | --- |
|  |  |  | Q1 | Q2 | Q3 | Q4 | Q5 |  |
| Participants, n |  | 5895 | 1180 | 1178 | 1179 | 1179 | 1179 |  |
| Age (years) |  | 57.5 (14.1) | 56.5 (14.5) | 56.8 (14.3) | 59.2 (13.7) | 59.3 (13.7) | 55.8 (13.8) | 0.45 |
| Sex | Men | 29.6 (1744) | 33.8 ( 399) | 29.8 ( 351) | 30.4 ( 358) | 28.4 ( 335) | 25.5 ( 301) | < 0.001 |
| BMI (kg/m^2^) |  | 23.1 (3.4) | 23.3 (3.6) | 23.0 (3.4) | 23.3 (3.4) | 23.0 (3.3) | 23.0 (3.4) | 0.025 |
| Morning home SBP (mmHg) |  | 125.0 (16.9) | 124.6 (17.0) | 124.4 (17.3) | 125.7 (16.1) | 125.9 (16.8) | 124.2 (17.1) | 0.53 |
| Morning home DBP (mmHg) |  | 75.0 (10.1) | 75.2 (9.9) | 74.6 (10.4) | 75.5 (10.0) | 75.0 (9.9) | 74.7 (10.2) | 0.60 |
| Home HT* | Yes | 38.9 (2296) | 38.7 ( 457) | 37.5 ( 442) | 42.1 ( 496) | 41.7 ( 492) | 34.7 ( 409) | 0.39 |
| Treatment for HT | Yes | 20.4 (1200) | 22.3 ( 263) | 18.3 ( 215) | 23.7 ( 279) | 20.8 ( 245) | 16.8 ( 198) | 0.023 |
| Household income | < 2 million yen | 11.6 ( 684) | 13.6 ( 161) | 11.0 ( 130) | 11.6 ( 137) | 10.7 ( 126) | 11.0 ( 130) | 0.058 |
|  | 2 to < 4 million yen | 39.3 (2316) | 33.1 ( 390) | 39.6 ( 466) | 44.7 ( 527) | 42.7 ( 504) | 36.4 ( 429) | 0.028 |
|  | 4 to < 6 million yen | 23.7 (1400) | 25.2 ( 297) | 23.1 ( 272) | 20.8 ( 245) | 23.5 ( 277) | 26.2 ( 309) | 0.53 |
|  | ≥ 6 million yen | 25.4 (1495) | 28.1 ( 332) | 26.3 ( 310) | 22.9 ( 270) | 23.1 ( 272) | 26.4 ( 311) | 0.092 |
| Seasonality | Summer | 38.7 (2279) | 40.6 ( 479) | 40.2 ( 474) | 37.2 ( 438) | 37.4 ( 441) | 37.9 ( 447) | 0.068 |
|  | Winter | 33.0 (1944) | 30.8 ( 364) | 32.6 ( 384) | 34.6 ( 408) | 33.6 ( 396) | 33.2 ( 392) | 0.18 |
|  | Other | 28.4 (1672) | 28.6 ( 337) | 27.2 ( 320) | 28.2 ( 333) | 29.0 ( 342) | 28.8 ( 340) | 0.56 |
| Drinking status | Never | 48.5 (2861) | 45.2 ( 533) | 49.5 ( 583) | 48.0 ( 566) | 50.0 ( 590) | 50.0 ( 589) | 0.028 |
|  | Past | 2.3 ( 138) | 3.2 ( 38) | 2.0 ( 24) | 2.0 ( 23) | 2.0 ( 23) | 2.5 ( 30) | 0.30 |
|  | Current | 49.1 (2896) | 51.6 ( 609) | 48.5 ( 571) | 50.0 ( 590) | 48.0 ( 566) | 47.5 ( 560) | 0.059 |
| Smoking status | Never | 66.0 (3889) | 60.6 ( 715) | 65.8 ( 775) | 66.5 ( 784) | 68.7 ( 810) | 68.3 ( 805) | < 0.001 |
|  | Past | 25.9 (1526) | 30.3 ( 358) | 26.4 ( 311) | 26.0 ( 306) | 24.5 ( 289) | 22.2 ( 262) | < 0.001 |
|  | Current | 8.1 ( 480) | 9.1 ( 107) | 7.8 ( 92) | 7.5 ( 89) | 6.8 ( 80) | 9.5 ( 112) | 0.95 |
| Morning urinary Na/K ratio |  | 4.7 (1.9) | 4.6 (1.8) | 4.7 (1.9) | 4.7 (2.0) | 4.8 (2.0) | 5.0 (2.1) | < 0.001 |
| Total wear time (min/day) |  | 907.9 (95.7) | 896.9 (95.5) | 906.7 (96.3) | 906.0 (95.7) | 912.5 (94.5) | 917.3 (95.5) | < 0.001 |
| Total PA-Acc (METs-h/day) |  | 25.9 (3.9) | 24.4 (3.6) | 25.6 (3.7) | 25.8 (3.8) | 26.5 (3.7) | 27.2 (3.9) | < 0.001 |
| Total PA-SR (METs-h/day) |  | 41.4 (13.7) | 26.5 (1.8) | 31.6 (1.7) | 37.7 (2.0) | 47.6 (3.6) | 63.8 (6.5) | < 0.001 |
| MVPA (min/day) |  | 61.1 (34.9) | 48.9 (26.8) | 57.9 (29.5) | 61.2 (34.3) | 65.4 (36.2) | 72.0 (41.4) | < 0.001 |
| LPA (min/day) |  | 385.3 (95.6) | 349.0 (95.7) | 378.6 (95.0) | 382.3 (91.5) | 399.3 (90.7) | 417.2 (91.6) | < 0.001 |
| SB (min/day) |  | 461.5 (110.0) | 499.0 (114.1) | 470.2 (107.7) | 462.6 (107.3) | 447.7 (102.3) | 428.1 (105.6) | < 0.001 |
| Steps (steps/day) |  | 6178.9 (2728.7) | 5334.4 (2417.1) | 6083.0 (2448.4) | 6151.6 (2665.5) | 6496.6 (2779.8) | 6829.4 (3055.3) | < 0.001 |

BMI, body mass index; SBP, systolic blood pressure; DBP, diastolic blood pressure; HT, hypertension; total PA, total physical activity; METs, metabolic equivalents; MVPA, moderate- to vigorous-intensity physical activity; SB, sedentary behavior; LPA, light-intensity physical activity; Acc, accelerometer-measured; SR, self-reported; Na/K ratio, Sodium-to-potassium ratio

^*^Home HT was defined as morning home SBP ≥135 mmHg and/or DBP ≥85 mmHg or receiving treatment for hypertension
