# Supplementary material for: Association of subjective and objective physical activity with home hypertension
Source: Hypertens Res. 2026 Feb 24;49(5):1586–96. doi: 10.1038/s41440-026-02587-8 (PMC13148978; doi:10.1038/s41440-026-02587-8)
Supplement: Supplementary file 3 — Supplementary Table 2 [file 41440_2026_2587_MOESM3_ESM.docx]

**Supplementary Table 2: Baseline characteristics of the participants according to MVPA**

|  |  | Overall | MVPA |  |  |  |  | *P* for trend |
| --- | --- | --- | --- | --- | --- | --- | --- | --- |
|  |  |  | Q1 | Q2 | Q3 | Q4 | Q5 |  |
| Participants, n |  | 5895 | 1179 | 1182 | 1180 | 1175 | 1179 |  |
| Age (years) |  | 57.5 (14.1) | 61.3 (14.2) | 57.5 (14.5) | 55.4 (14.4) | 56.3 (13.9) | 57.1 (12.5) | < 0.001 |
| Sex | Men | 29.6 (1744) | 38.9 ( 459) | 30.1 ( 356) | 24.6 ( 290) | 26.9 ( 316) | 27.4 ( 323) | < 0.001 |
| BMI (kg/m^2^) |  | 23.1 (3.4) | 23.5 (3.5) | 23.3 (3.7) | 23.0 (3.5) | 22.9 (3.2) | 22.8 (3.1) | < 0.001 |
| Morning home SBP (mmHg) |  | 125.0 (16.9) | 127.2 (17.5) | 125.5 (17.2) | 123.4 (16.6) | 124.2 (16.6) | 124.4 (16.2) | < 0.001 |
| Morning home DBP (mmHg) |  | 75.0 (10.1) | 75.4 (10.2) | 75.3 (9.9) | 74.5 (10.1) | 74.8 (10.2) | 74.9 (10.1) | 0.086 |
| Home HT* | Yes | 38.9 (2296) | 48.4 ( 571) | 40.7 ( 481) | 35.5 ( 419) | 34.4 ( 404) | 35.7 ( 421) | < 0.001 |
| Treatment for HT | Yes | 20.4 (1200) | 28.9 ( 341) | 21.0 ( 248) | 17.9 ( 211) | 17.3 ( 203) | 16.7 ( 197) | < 0.001 |
| Household income | < 2 million yen | 11.6 ( 684) | 13.6 ( 160) | 13.0 ( 154) | 10.8 ( 128) | 11.1 ( 131) | 9.4 ( 111) | < 0.001 |
|  | 2 to < 4 million yen | 39.3 (2316) | 42.5 ( 501) | 36.0 ( 425) | 38.8 ( 458) | 40.0 ( 470) | 39.2 ( 462) | 0.57 |
|  | 4 to < 6 million yen | 23.7 (1400) | 22.5 ( 265) | 26.1 ( 309) | 22.6 ( 267) | 21.4 ( 251) | 26.1 ( 308) | 0.52 |
|  | ≥ 6 million yen | 25.4 (1495) | 21.5 ( 253) | 24.9 ( 294) | 27.7 ( 327) | 27.5 ( 323) | 25.3 ( 298) | 0.011 |
| Seasonality | Summer | 38.7 (2279) | 38.0 ( 448) | 37.0 ( 437) | 39.6 ( 467) | 38.0 ( 446) | 40.8 ( 481) | 0.14 |
|  | Winter | 33.0 (1944) | 34.8 ( 410) | 36.0 ( 425) | 32.5 ( 383) | 31.5 ( 370) | 30.2 ( 356) | 0.0017 |
|  | Other | 28.4 (1672) | 27.2 ( 321) | 27.1 ( 320) | 28.0 ( 330) | 30.6 ( 359) | 29.0 ( 342) | 0.090 |
| Drinking status | Never | 48.5 (2861) | 48.1 ( 567) | 48.8 ( 577) | 50.5 ( 596) | 48.4 ( 569) | 46.8 ( 552) | 0.52 |
|  | Past | 2.3 ( 138) | 3.4 ( 40) | 2.2 ( 26) | 2.5 ( 30) | 2.0 ( 23) | 1.6 ( 19) | 0.0066 |
|  | Current | 49.1 (2896) | 48.5 ( 572) | 49.0 ( 579) | 46.9 ( 554) | 49.6 ( 583) | 51.6 ( 608) | 0.14 |
| Smoking status | Never | 66.0 (3889) | 61.2 ( 721) | 64.6 ( 763) | 68.3 ( 806) | 66.0 ( 776) | 69.8 ( 823) | < 0.001 |
|  | Past | 25.9 (1526) | 27.7 ( 326) | 27.7 ( 327) | 24.5 ( 289) | 26.4 ( 310) | 23.2 ( 274) | 0.012 |
|  | Current | 8.1 ( 480) | 11.2 ( 132) | 7.8 ( 92) | 7.2 ( 85) | 7.6 ( 89) | 7.0 ( 82) | < 0.001 |
| Morning urinary Na/K ratio |  | 4.7 (1.9) | 4.9 (2.0) | 4.7 (1.9) | 4.6 (1.9) | 4.7 (1.9) | 4.7 (1.9) | 0.060 |
| Total wear time (min/day) |  | 907.9 (95.7) | 882.1 (94.5) | 897.2 (94.5) | 911.9 (93.6) | 918.1 (93.5) | 930.2 (95.2) | < 0.001 |
| Total PA-Acc (METs-h/day) |  | 25.9 (3.9) | 22.5 (2.9) | 24.3 (2.8) | 25.9 (2.8) | 27.1 (2.8) | 29.8 (3.4) | < 0.001 |
| Total PA-SR (METs-h/day) |  | 41.4 (13.7) | 37.9 (12.9) | 39.7 (12.6) | 41.0 (13.6) | 43.0 (13.8) | 45.6 (14.2) | < 0.001 |
| MVPA (min/day) |  | 61.1 (34.9) | 22.6 (7.8) | 40.6 (4.3) | 55.0 (4.3) | 72.6 (6.1) | 114.8 (31.6) | < 0.001 |
| LPA (min/day) |  | 385.3 (95.6) | 335.8 (92.5) | 365.3 (86.9) | 394.2 (89.5) | 405.3 (91.2) | 425.8 (91.4) | < 0.001 |
| SB (min/day) |  | 461.5 (110.0) | 523.7 (106.5) | 491.3 (99.1) | 462.6 (99.5) | 440.2 (99.6) | 389.6 (95.8) | < 0.001 |
| Steps (steps/day) |  | 6178.9 (2728.7) | 3559.7 (1329.3) | 4990.6 (1409.5) | 6031.3 (1569.0) | 7127.1 (1906.5) | 9192.1 (3019.7) | < 0.001 |

BMI, body mass index; SBP, systolic blood pressure; DBP, diastolic blood pressure; HT, hypertension; total PA, total physical activity; METs, metabolic equivalents; MVPA, moderate- to vigorous-intensity physical activity; SB, sedentary behavior; LPA, light-intensity physical activity; Acc, accelerometer-measured; SR, self-reported; Na/K ratio, Sodium-to-potassium ratio

^*^Home HT was defined as morning home SBP ≥135 mmHg and/or DBP ≥85 mmHg or receiving treatment for hypertension
