# Supplementary material for: Association of subjective and objective physical activity with home hypertension
Source: Hypertens Res. 2026 Feb 24;49(5):1586–96. doi: 10.1038/s41440-026-02587-8 (PMC13148978; doi:10.1038/s41440-026-02587-8)
Supplement: Supplementary file 4 — Supplementary Table 3 [file 41440_2026_2587_MOESM4_ESM.docx]

**Supplementary Table 3: Baseline characteristics of the participants according to LPA**

|  |  | Overall | LPA |  |  |  |  | *P* for trend |
| --- | --- | --- | --- | --- | --- | --- | --- | --- |
|  |  |  | Q1 | Q2 | Q3 | Q4 | Q5 |  |
| Participants, n |  | 5895 | 1180 | 1178 | 1179 | 1179 | 1179 |  |
| Age (years) |  | 57.5 (14.1) | 61.5 (13.8) | 59.2 (14.0) | 56.3 (14.3) | 55.4 (14.1) | 55.2 (13.0) | < 0.001 |
| Sex | Men | 29.6 (1744) | 65.5 ( 773) | 37.1 ( 437) | 21.8 ( 257) | 15.4 ( 182) | 8.1 ( 95) | < 0.001 |
| BMI (kg/m^2^) |  | 23.1 (3.4) | 24.0 (3.4) | 23.6 (3.4) | 23.0 (3.4) | 22.8 (3.3) | 22.2 (3.2) | < 0.001 |
| Morning home SBP (mmHg) |  | 125.0 (16.9) | 129.9 (16.7) | 126.5 (16.5) | 124.0 (16.8) | 122.7 (16.4) | 121.7 (16.8) | < 0.001 |
| Morning home DBP (mmHg) |  | 75.0 (10.1) | 77.5 (10.0) | 75.8 (10.0) | 74.6 (10.0) | 74.0 (9.8) | 73.0 (10.1) | < 0.001 |
| Home HT* | Yes | 38.9 (2296) | 53.6 ( 632) | 42.5 ( 501) | 36.1 ( 426) | 33.9 ( 400) | 28.6 ( 337) | < 0.001 |
| Treatment for HT | Yes | 20.4 (1200) | 30.9 ( 365) | 22.3 ( 263) | 17.9 ( 211) | 17.0 ( 201) | 13.6 ( 160) | < 0.001 |
| Household income | < 2 million yen | 11.6 ( 684) | 13.9 ( 164) | 12.5 ( 147) | 10.9 ( 128) | 12.1 ( 143) | 8.7 ( 102) | < 0.001 |
|  | 2 to < 4 million yen | 39.3 (2316) | 42.7 ( 504) | 41.3 ( 486) | 37.2 ( 439) | 36.2 ( 427) | 39.0 ( 460) | 0.0057 |
|  | 4 to < 6 million yen | 23.7 (1400) | 20.8 ( 246) | 23.5 ( 277) | 23.4 ( 276) | 25.8 ( 304) | 25.2 ( 297) | 0.0052 |
|  | ≥ 6 million yen | 25.4 (1495) | 22.5 ( 266) | 22.8 ( 268) | 28.5 ( 336) | 25.9 ( 305) | 27.1 ( 320) | 0.0021 |
| Seasonality | Summer | 38.7 (2279) | 33.9 ( 400) | 35.7 ( 420) | 38.3 ( 452) | 40.8 ( 481) | 44.6 ( 526) | < 0.001 |
|  | Winter | 33.0 (1944) | 40.7 ( 480) | 35.0 ( 412) | 33.4 ( 394) | 29.9 ( 353) | 25.9 ( 305) | < 0.001 |
|  | Other | 28.4 (1672) | 25.4 ( 300) | 29.4 ( 346) | 28.2 ( 333) | 29.3 ( 345) | 29.5 ( 348) | 0.052 |
| Drinking status | Never | 48.5 (2861) | 37.2 ( 439) | 45.3 ( 534) | 51.4 ( 606) | 52.8 ( 623) | 55.9 ( 659) | < 0.001 |
|  | Past | 2.3 ( 138) | 2.8 ( 33) | 1.9 ( 22) | 2.9 ( 34) | 2.5 ( 29) | 1.7 ( 20) | 0.25 |
|  | Current | 49.1 (2896) | 60.0 ( 708) | 52.8 ( 622) | 45.7 ( 539) | 44.7 ( 527) | 42.4 ( 500) | < 0.001 |
| Smoking status | Never | 66.0 (3889) | 47.5 ( 560) | 62.7 ( 739) | 68.7 ( 810) | 72.9 ( 859) | 78.1 ( 921) | < 0.001 |
|  | Past | 25.9 (1526) | 39.4 ( 465) | 29.4 ( 346) | 23.2 ( 273) | 21.0 ( 248) | 16.5 ( 194) | < 0.001 |
|  | Current | 8.1 ( 480) | 13.1 ( 155) | 7.9 ( 93) | 8.1 ( 96) | 6.1 ( 72) | 5.4 ( 64) | < 0.001 |
| Morning urinary Na/K ratio |  | 4.7 (1.9) | 4.8 (1.9) | 4.6 (2.0) | 4.8 (2.0) | 4.7 (1.9) | 4.7 (2.0) | 0.86 |
| Total wear time (min/day) |  | 907.9 (95.7) | 848.9 (89.9) | 884.8 (87.4) | 906.4 (88.1) | 925.0 (82.7) | 974.4 (82.4) | < 0.001 |
| Total PA-Acc (METs-h/day) |  | 25.9 (3.9) | 21.6 (2.9) | 24.3 (2.3) | 25.9 (2.4) | 27.6 (2.4) | 30.2 (2.7) | < 0.001 |
| Total PA-SR (METs-h/day) |  | 41.4 (13.7) | 36.7 (11.6) | 39.3 (12.6) | 42.1 (13.6) | 43.4 (13.9) | 45.7 (14.8) | < 0.001 |
| MVPA (min/day) |  | 61.1 (34.9) | 46.6 (31.9) | 55.6 (31.9) | 59.8 (32.9) | 68.9 (34.9) | 74.6 (35.6) | < 0.001 |
| LPA (min/day) |  | 385.3 (95.6) | 249.6 (42.8) | 334.0 (16.6) | 387.2 (14.4) | 438.0 (16.2) | 517.6 (42.6) | < 0.001 |
| SB (min/day) |  | 461.5 (110.0) | 552.7 (99.7) | 495.2 (94.6) | 459.3 (93.6) | 418.1 (88.4) | 382.2 (86.2) | < 0.001 |
| Steps (steps/day) |  | 6178.9 (2728.7) | 5307.4 (2989.0) | 5916.4 (2711.7) | 6114.1 (2453.0) | 6569.3 (2545.3) | 6987.7 (2613.1) | < 0.001 |

BMI, body mass index; SBP, systolic blood pressure; DBP, diastolic blood pressure; HT, hypertension; total PA, total physical activity; METs, metabolic equivalents; MVPA, moderate- to vigorous-intensity physical activity; SB, sedentary behavior; LPA, light-intensity physical activity; Acc, accelerometer-measured; SR, self-reported; Na/K ratio, Sodium-to-potassium ratio

^*^Home HT was defined as morning home SBP ≥135 mmHg and/or DBP ≥85 mmHg or receiving treatment for hypertension
