# Supplementary material for: Association of subjective and objective physical activity with home hypertension
Source: Hypertens Res. 2026 Feb 24;49(5):1586–96. doi: 10.1038/s41440-026-02587-8 (PMC13148978; doi:10.1038/s41440-026-02587-8)
Supplement: Supplementary file 5 — Supplementary Table 4 [file 41440_2026_2587_MOESM5_ESM.docx]

**Supplementary Table 4: Baseline characteristics of the participants according to SB**

|  |  | Overall | SB |  |  |  |  | *P* for trend |
| --- | --- | --- | --- | --- | --- | --- | --- | --- |
|  |  |  | Q1 | Q2 | Q3 | Q4 | Q5 |  |
| Participants, n |  | 5895 | 1179 | 1182 | 1178 | 1177 | 1179 |  |
| Age (years) |  | 57.5 (14.1) | 54.0 (14.7) | 57.0 (14.2) | 58.0 (14.1) | 59.5 (13.3) | 59.2 (13.4) | < 0.001 |
| Sex | Men | 29.6 (1744) | 21.9 ( 258) | 22.5 ( 266) | 28.9 ( 341) | 32.6 ( 384) | 42.0 ( 495) | < 0.001 |
| BMI (kg/m^2^) |  | 23.1 (3.4) | 22.8 (3.3) | 22.8 (3.3) | 23.0 (3.4) | 23.3 (3.4) | 23.6 (3.6) | < 0.001 |
| Morning home SBP (mmHg) |  | 125.0 (16.9) | 123.6 (17.4) | 123.7 (16.8) | 125.0 (16.6) | 126.1 (16.7) | 126.4 (16.7) | < 0.001 |
| Morning home DBP (mmHg) |  | 75.0 (10.1) | 74.3 (10.5) | 74.3 (10.1) | 74.7 (9.8) | 75.5 (9.9) | 76.2 (10.1) | < 0.001 |
| Home HT* | Yes | 38.9 (2296) | 34.2 ( 403) | 35.8 ( 423) | 38.8 ( 457) | 42.1 ( 495) | 43.9 ( 518) | < 0.001 |
| Treatment for HT | Yes | 20.4 (1200) | 15.5 ( 183) | 19.0 ( 224) | 20.5 ( 241) | 23.3 ( 274) | 23.6 ( 278) | < 0.001 |
| Household income | < 2 million yen | 11.6 ( 684) | 10.6 ( 125) | 11.5 ( 136) | 12.1 ( 142) | 12.1 ( 143) | 11.7 ( 138) | 0.33 |
|  | 2 to < 4 million yen | 39.3 (2316) | 39.4 ( 464) | 39.8 ( 470) | 41.9 ( 493) | 38.0 ( 447) | 37.5 ( 442) | 0.22 |
|  | 4 to < 6 million yen | 23.7 (1400) | 25.6 ( 302) | 23.4 ( 277) | 22.2 ( 261) | 22.3 ( 262) | 25.3 ( 298) | 0.64 |
|  | ≥ 6 million yen | 25.4 (1495) | 24.4 ( 288) | 25.3 ( 299) | 23.9 ( 282) | 27.6 ( 325) | 25.5 ( 301) | 0.26 |
| Seasonality | Summer | 38.7 (2279) | 41.1 ( 485) | 40.1 ( 474) | 37.1 ( 437) | 36.4 ( 429) | 38.5 ( 454) | 0.047 |
|  | Winter | 33.0 (1944) | 28.2 ( 332) | 29.9 ( 353) | 35.7 ( 420) | 35.3 ( 415) | 36.0 ( 424) | < 0.001 |
|  | Other | 28.4 (1672) | 30.7 ( 362) | 30.0 ( 355) | 27.2 ( 321) | 28.3 ( 333) | 25.5 ( 301) | 0.0036 |
| Drinking status | Never | 48.5 (2861) | 49.7 ( 586) | 51.4 ( 607) | 49.0 ( 577) | 46.8 ( 551) | 45.8 ( 540) | 0.0073 |
|  | Past | 2.3 ( 138) | 2.5 ( 29) | 2.8 ( 33) | 2.3 ( 27) | 1.3 ( 15) | 2.9 ( 34) | 0.63 |
|  | Current | 49.1 (2896) | 47.8 ( 564) | 45.9 ( 542) | 48.7 ( 574) | 51.9 ( 611) | 51.3 ( 605) | 0.0047 |
| Smoking status | Never | 66.0 (3889) | 67.6 ( 797) | 70.0 ( 827) | 66.5 ( 783) | 65.4 ( 770) | 60.4 ( 712) | < 0.001 |
|  | Past | 25.9 (1526) | 24.3 ( 286) | 22.3 ( 263) | 26.7 ( 315) | 27.1 ( 319) | 29.1 ( 343) | < 0.001 |
|  | Current | 8.1 ( 480) | 8.1 ( 96) | 7.8 ( 92) | 6.8 ( 80) | 7.5 ( 88) | 10.5 ( 124) | 0.078 |
| Morning urinary Na/K ratio |  | 4.7 (1.9) | 4.8 (2.0) | 4.8 (1.9) | 4.7 (2.0) | 4.7 (2.0) | 4.6 (1.9) | 0.0061 |
| Total wear time (min/day) |  | 907.9 (95.7) | 854.7 (88.2) | 892.5 (89.8) | 903.3 (85.5) | 922.3 (84.0) | 966.6 (94.1) | < 0.001 |
| Total PA-Acc (METs-h/day) |  | 25.9 (3.9) | 27.5 (3.8) | 26.7 (3.9) | 25.7 (3.6) | 25.1 (3.5) | 24.5 (3.7) | < 0.001 |
| Total PA-SR (METs-h/day) |  | 41.4 (13.7) | 45.0 (14.5) | 43.2 (13.7) | 42.4 (13.9) | 39.5 (12.7) | 37.1 (12.2) | < 0.001 |
| MVPA (min/day) |  | 61.1 (34.9) | 84.3 (42.1) | 68.1 (34.5) | 59.2 (29.1) | 51.3 (24.9) | 42.5 (25.0) | < 0.001 |
| LPA (min/day) |  | 385.3 (95.6) | 457.3 (77.3) | 423.8 (82.0) | 386.5 (78.1) | 353.8 (78.1) | 304.8 (81.9) | < 0.001 |
| SB (min/day) |  | 461.5 (110.0) | 313.0 (46.3) | 400.7 (18.7) | 457.6 (15.6) | 517.2 (19.1) | 619.3 (60.2) | < 0.001 |
| Steps (steps/day) |  | 6178.9 (2728.7) | 7417.5 (2937.0) | 6655.3 (2708.5) | 6159.2 (2451.9) | 5700.2 (2427.6) | 4960.2 (2429.8) | < 0.001 |

BMI, body mass index; SBP, systolic blood pressure; DBP, diastolic blood pressure; HT, hypertension; total PA, total physical activity; METs, metabolic equivalents; MVPA, moderate- to vigorous-intensity physical activity; SB, sedentary behavior; LPA, light-intensity physical activity; Acc, accelerometer-measured; SR, self-reported; Na/K ratio, Sodium-to-potassium ratio

^*^Home HT was defined as morning home SBP ≥135 mmHg and/or DBP ≥85 mmHg or receiving treatment for hypertension
