# Supplementary material for: Association of subjective and objective physical activity with home hypertension
Source: Hypertens Res. 2026 Feb 24;49(5):1586–96. doi: 10.1038/s41440-026-02587-8 (PMC13148978; doi:10.1038/s41440-026-02587-8)
Supplement: Supplementary file 6 — Supplementary Table 5 [file 41440_2026_2587_MOESM6_ESM.docx]

**Supplementary Table 5: Baseline characteristics of the participants according to steps**

|  |  | Overall | Steps |  |  |  |  | *P* for trend |
| --- | --- | --- | --- | --- | --- | --- | --- | --- |
|  |  |  | Q1 | Q2 | Q3 | Q4 | Q5 |  |
| Participants, n |  | 5895 | 1179 | 1179 | 1179 | 1179 | 1179 |  |
| Age (years) |  | 57.5 (14.1) | 61.9 (13.6) | 56.8 (14.4) | 56.6 (14.1) | 55.9 (13.8) | 56.4 (13.7) | < 0.001 |
| Sex | Men | 29.6 (1744) | 29.8 ( 351) | 23.7 ( 279) | 27.4 ( 323) | 28.3 ( 334) | 38.8 ( 457) | < 0.001 |
| BMI (kg/m^2^) |  | 23.1 (3.4) | 23.8 (3.9) | 23.2 (3.5) | 22.9 (3.3) | 22.8 (3.3) | 22.9 (3.0) | < 0.001 |
| Morning home SBP (mmHg) |  | 125.0 (16.9) | 128.2 (17.3) | 124.0 (17.2) | 123.6 (16.8) | 124.0 (16.5) | 124.9 (16.1) | < 0.001 |
| Morning home DBP (mmHg) |  | 75.0 (10.1) | 75.7 (10.3) | 74.5 (10.2) | 74.5 (9.9) | 74.9 (10.0) | 75.5 (10.0) | 0.99 |
| Home HT* | Yes | 38.9 (2296) | 48.8 ( 575) | 38.6 ( 455) | 36.6 ( 431) | 34.8 ( 410) | 36.0 ( 425) | < 0.001 |
| Treatment for HT | Yes | 20.4 (1200) | 28.1 ( 331) | 21.0 ( 248) | 18.7 ( 220) | 17.2 ( 203) | 16.8 ( 198) | < 0.001 |
| Household income | < 2 million yen | 11.6 ( 684) | 16.1 ( 190) | 11.8 ( 139) | 11.2 ( 132) | 8.8 ( 104) | 10.1 ( 119) | < 0.001 |
|  | 2 to < 4 million yen | 39.3 (2316) | 42.6 ( 502) | 39.0 ( 460) | 37.7 ( 444) | 39.3 ( 463) | 37.9 ( 447) | 0.044 |
|  | 4 to < 6 million yen | 23.7 (1400) | 22.6 ( 266) | 24.4 ( 288) | 22.5 ( 265) | 23.5 ( 277) | 25.8 ( 304) | 0.16 |
|  | ≥ 6 million yen | 25.4 (1495) | 18.7 ( 221) | 24.8 ( 292) | 28.7 ( 338) | 28.4 ( 335) | 26.2 ( 309) | < 0.001 |
| Seasonality | Summer | 38.7 (2279) | 31.8 ( 375) | 38.9 ( 459) | 39.3 ( 463) | 39.9 ( 471) | 43.3 ( 511) | < 0.001 |
|  | Winter | 33.0 (1944) | 42.2 ( 498) | 34.7 ( 409) | 32.1 ( 378) | 29.2 ( 344) | 26.7 ( 315) | < 0.001 |
|  | Other | 28.4 (1672) | 26.0 ( 306) | 26.4 ( 311) | 28.7 ( 338) | 30.9 ( 364) | 29.9 ( 353) | 0.0027 |
| Drinking status | Never | 48.5 (2861) | 52.6 ( 620) | 52.0 ( 613) | 48.0 ( 566) | 44.7 ( 527) | 45.4 ( 535) | < 0.001 |
|  | Past | 2.3 ( 138) | 3.6 ( 42) | 2.3 ( 27) | 2.0 ( 24) | 1.9 ( 22) | 2.0 ( 23) | 0.0092 |
|  | Current | 49.1 (2896) | 43.9 ( 517) | 45.7 ( 539) | 50.0 ( 589) | 53.4 ( 630) | 52.7 ( 621) | < 0.001 |
| Smoking status | Never | 66.0 (3889) | 65.3 ( 770) | 66.2 ( 781) | 65.7 ( 775) | 67.2 ( 792) | 65.4 ( 771) | 0.80 |
|  | Past | 25.9 (1526) | 25.2 ( 297) | 25.4 ( 300) | 27.3 ( 322) | 25.4 ( 300) | 26.0 ( 307) | 0.67 |
|  | Current | 8.1 ( 480) | 9.5 ( 112) | 8.3 ( 98) | 7.0 ( 82) | 7.4 ( 87) | 8.6 ( 101) | 0.27 |
| Morning urinary Na/K ratio |  | 4.7 (1.9) | 4.8 (2.0) | 4.7 (2.0) | 4.7 (1.9) | 4.7 (1.9) | 4.8 (1.9) | 0.95 |
| Total wear time (min/day) |  | 907.9 (95.7) | 888.2 (95.7) | 900.8 (96.9) | 908.7 (92.5) | 921.6 (94.1) | 920.1 (95.3) | < 0.001 |
| Total PA-Acc (METs-h/day) |  | 25.9 (3.9) | 23.0 (3.3) | 25.0 (3.2) | 25.9 (3.3) | 27.1 (3.4) | 28.6 (3.7) | < 0.001 |
| Total PA-SR (METs-h/day) |  | 41.4 (13.7) | 37.9 (12.7) | 40.4 (13.1) | 41.5 (13.7) | 42.2 (13.7) | 45.2 (14.3) | < 0.001 |
| MVPA (min/day) |  | 61.1 (34.9) | 30.4 (18.4) | 45.5 (18.5) | 58.3 (22.0) | 71.2 (25.2) | 100.0 (38.4) | < 0.001 |
| LPA (min/day) |  | 385.3 (95.6) | 333.1 (89.4) | 385.0 (89.0) | 393.4 (91.2) | 407.7 (91.6) | 407.1 (96.8) | < 0.001 |
| SB (min/day) |  | 461.5 (110.0) | 524.7 (108.0) | 470.3 (101.8) | 456.9 (100.7) | 442.7 (100.2) | 413.0 (107.3) | < 0.001 |
| Steps (steps/day) |  | 6178.9 (2728.7) | 2971.9 (783.8) | 4611.3 (361.0) | 5799.0 (351.8) | 7203.2 (480.6) | 10309.0 (2265.9) | < 0.001 |

BMI, body mass index; SBP, systolic blood pressure; DBP, diastolic blood pressure; HT, hypertension; total PA, total physical activity; METs, metabolic equivalents; MVPA, moderate- to vigorous-intensity physical activity; SB, sedentary behavior; LPA, light-intensity physical activity; Acc, accelerometer-measured; SR, self-reported; Na/K ratio, Sodium-to-potassium ratio

^*^Home HT was defined as morning home SBP ≥135 mmHg and/or DBP ≥85 mmHg or receiving treatment for hypertension
