# Supplementary material for: Association of subjective and objective physical activity with home hypertension
Source: Hypertens Res. 2026 Feb 24;49(5):1586–96. doi: 10.1038/s41440-026-02587-8 (PMC13148978; doi:10.1038/s41440-026-02587-8)
Supplement: Supplementary file 7 — Supplementary Table 6 [file 41440_2026_2587_MOESM7_ESM.docx]

**Supplementary Table 6: Multiple regression analysis of the association between total PA-Acc and total PA-SR**

|  | β | 95% CI lower | 95% CI upper | *P*-value |
| --- | --- | --- | --- | --- |
| Total PA-Acc | 0.902 | 0.808 | 0.996 | < 0.001 |
| Sex (Men) | 0.866 | 0.0533 | 1.68 | 0.0368 |
| Age | -0.00516 | -0.0317 | 0.0214 | 0.704 |
| Household income (2 to < 4 million yen) | 0.362 | -0.777 | 1.50 | 0.533 |
| Household income (4 to < 6 million yen) | 0.262 | -0.988 | 1.51 | 0.682 |
| Household income (≥ 6 million yen) | -0.386 | -1.64 | 0.865 | 0.545 |

Total PA, total physical activity; Acc, accelerometer-measured; SR, self-reported; CI, confidence interval
